# Migration patterns & their associations with health and human rights in eastern Myanmar after political transition: results of a population-based survey using multistaged household cluster sampling

**DOI:** 10.1186/s13031-019-0193-1

**Published:** 2019-04-27

**Authors:** Parveen K. Parmar, Charlene Barina, Sharon Low, Kyaw Thura Tun, Conrad Otterness, Pue P. Mhote, Saw Nay Htoo, Saw Win Kyaw, Nai Aye Lwin, Cynthia Maung, Naw Merry Moo, Eh. Kalu Shwee Oo, Daniel Reh, Nai Chay Mon, Xinkai Zhou, Adam K. Richards

**Affiliations:** 1 Division of Global Emergency Medicine, Department of Emergency Medicine, University of Southern California, 1975 Zonal Ave, Los Angeles, CA 90033 USA; 2 Community Partners International, 2550 Ninth St. Suite 111, Berkeley, CA 94710 USA; 3 Present Address: Clinton Health Access Initiative, Hanoi, Vietnam; 4 Present Address: Washington State Department of Health, Tumwater, USA; 5 Burma Medical Association (BMA), Mae Sot, Thailand; 6 Health Information Systems Information Group (HISWG), Mae Sot, Thailand; 7 Back Pack Health Worker Team, Mae Sot, Thailand; 8 Mae Tao Clinic, Mae Sot, Thailand; 9 Karen Department of Health and Welfare, Hpa-An, Myanmar; 10 Karenni Mobile Health Committee (KnMHC), Mae Hong Son, Thailand; 11 Mon National Health Committee (MNHC), Sangkhlaburi, Thailand; 12 Department of Medicine, Statistics Core, David Geffen School of Medicine at University of California, Los Angeles, USA; 13 Division of General Internal Medicine and Health Services Research, University of California, Los Angeles, USA; 14 International organization for Migration, Mogadishu, Somalia

**Keywords:** Myanmar, Migration, Health, Human rights

## Abstract

**Background:**

Myanmar transitioned to a nominally civilian government in March 2011. It is unclear how, if at all, this political change has impacted migration at the household level.

**Methods:**

We present household-level in- and out-migration data gathered during the Eastern Burma Retrospective Mortality Survey (EBRMS) conducted in 2013. Household level in-and out-migration information within the previous year was gathered via a cross-sectional, retrospective, multi-stage population-based cluster randomized survey conducted in eastern Myanmar. Univariate, bivariate and regression analyses were conducted.

**Results:**

We conducted a cross-sectional survey of 6620 households across Eastern Myanmar between July and September of 2013. Out-migration outstripped in-migration more than 6:1 overall during the year prior to the survey – for international migration this ratio was 29:1. Most in-migrants had moved to their present location in the study area from other areas in Myanmar (87%). Only 11.3% (27 individuals) had returned from another country (Thailand). Those who migrated out of eastern Myanmar during the previous year were more likely to be male (55.2%), and three times more likely to be between the ages of 15–25 (49.5%) than non-migrants. The primary reason cited for a return to the household was family (26.3%) followed by work (23.2%). The primary reason cited for migrating out of the household was for education (46.4%) followed by work (40.2%). Respondents from households that reported out-migration in the past year were more likely to screen positive for depressive symptoms than households with no migration (PR 1.85; 95% CI 1.16, 2.97). Women in households with in-migration were more likely to be malnourished and had a higher unmet need for contraception. Forced labor, one subset of human rights violations experienced by this population, was reported by more in-migrant (8%) than out-migrant households (2.2%), though this finding did not reach statistical significance.

**Conclusions:**

These analyses suggest that opportunities for employment and education are the primary drivers of migration out of the household, despite an overall improvement in stability and decrease in prevalence of human rights violations found by EBRMS 2013. Additionally, migration into and out of households in eastern Myanmar is associated with changes in health outcomes.

## Background

Burma/Myanmar (hereafter Myanmar) transitioned to a nominally civilian government in March 2011. In early 2016 the National League for Democracy (NLD) took enough seats in parliament to form a government and installed the nation’s first civilian president in 53 years [1]. These developments, as well as nationwide efforts to formalize ceasefire agreements with armed ethnic groups have led to an environment of moderate reforms and gradually improving security. While there have been increases in humanitarian aid and business investments [2, 3], conflict and poor health indicators still persist [4–7]. In this complex context, discussion of refugee and migrant return to Myanmar has begun [8, 9].

The International Organization for Migration estimates that approximately 50–55 million people, or roughly 10% of Myanmar’s population, migrate internationally, each year [10]. Migration from Myanmar occurs for a host of reasons, and includes a spectrum of economic migrants to those displaced by conflict and human rights violations, including asylum seekers and refugees. Traditionally, “push” factors for migration out of Myanmar have included widespread poverty and lack of livelihoods, insecurity, human rights violations, and direct and indirect displacement by commercial and military development projects [11, 12]. “Pull” factors encouraging migration from Myanmar include employment opportunities with substantially higher wages, improved physical security, and access to services including health care and education [12]. Neighboring Thailand hosts an estimated 2–4 million migrants from Myanmar working in low-skilled and semi-skilled jobs including agriculture, domestic services, clothing production, construction, and fishing [13–15]. The Thai government has recognized the central role played by Burmese migrants in their economy, and has formalized a verification, registration, and recruitment process—providing further “pull” for potential migrants [15]. Additionally, the World Bank estimates that remittances from Thailand to Myanmar accounted for 150 million USD in 2008, which is largely thought to be an underestimate given the predominate use of informal money transfer mechanisms [16]. These remittances serve as an important resource for households in Myanmar, aiding with daily living expenses, health, housing, and education [16]. It is unclear how, if at all, these “push” and “pull” factors have changed, and how these factors affect migration at the household level after the political transition. Additionally, a large proportion of migrants move within the country, either inside their state/administrative region or across state/administrative region lines—most often to find employment opportunities [17], however many have historically migrated to avoid conflict or human rights violations, as internally displaced persons.

The Eastern Burma Retrospective Mortality Survey (EBRMS) was a large, population-based survey conducted in 2009 and 2013 in three states and two administrative regions^1^ in Eastern Myanmar. It explored demographics, mortality, health outcomes, water and sanitation, food security and nutrition, malaria, and human rights violations. This survey found that while human rights violations were less common in 2013 than in 2009, exposure to human rights violations at the household level was associated with a higher prevalence of moderate to severe malnutrition and increased prevalence of self-reported fair or poor health status [18].

Below we present household-level in- and out-migration data gathered during the Eastern Burma Retrospective Mortality Survey conducted in 2013. The aim was to gather data on the demographic composition of migrants, the frequency and primary drivers as well as geographic patterns of migration, and to explore associations of in- and out-migration with health and human rights outcomes.

## Methods

### Design

The survey design and descriptive results have been previously presented [18]. Briefly, 80 surveyors conducted a retrospective, cross-sectional household survey in five states and regions between July 2013–September 2013. Surveyed areas included accessible areas of Bago, Karen, Karenni, Tanintharyi, and Mon [4]. The primary objective of this study was to estimate morbidity and mortality in the service areas of five health community-based organizations (CBOs) that deliver services to internally displaced persons (IDPs) and other populations in eastern Myanmar. Additional studied outcomes included household migration, demographics, mortality, self-reported health status, child health, reproductive health, food access and nutrition, water and sanitation, human rights violations, malaria, and access to health services. The sampling frame of 456,786 people (87,841 households) was constructed using CBO provided village-level population lists updated within the year prior to the survey. Clusters were selected using probability proportional to size (PPS) in the first stage. In the second stage, proximity sampling was used to select 30 households for each cluster. A household was defined as a group of people who live under the same roof for two or more months and share meals.

### Implementation

The survey was written in English, translated to Burmese, Mon, and Sgaw Karen and back-translated from each respective local language into English. The survey asked respondents to enumerate the sex, age, and in−/out-migration of all household members. Additionally, respondents were asked to give the age and perceived cause of death of all who died in the household in the past year with the exception of miscarriages, abortions, and stillbirths. Heads of household (male or female) were asked to respond to the first 78 survey questions. When the head of household was not available, respondents were selected in the following descending order of priority: Women of reproductive age (15–49, WRA) with the youngest child under five in the household, WRA currently pregnant, oldest WRA.

### Migration

Survey respondents were asked to enumerate all individuals currently living in the household, as well as former household members who subsequently had moved away. In-migrants were defined as individuals who reportedly had lived in the household for at least 2 but no more than 12 months. A 2-month minimum residency period was used to exclude transient visitors. Out-migrants were defined as individuals who reportedly had lived in the household within the past 12 months but had subsequently left. Respondents were asked where each individual had moved to/from (within State; outside State but within Myanmar; to/from Thailand; to/from Malaysia; or to/from another country); and the main reason for having returned or moved away. International migrants are those who have crossed an international border. Out-migrants include those who previously lived in the interviewed household and now live in another community; and their new location could be in the same state in Myanmar; a different state of Myanmar; or another country. Household members who migrated into or out of a household greater than 12 months before the survey were categorized as “non-migrants” to minimize recall bias and to focus the study on the period after political transition. Information on age, sex, and relationship of each individual to the respondent was also collected.

### Health outcomes

The Patient Health Questionnaire-2 (PHQ-2), a 2-question screen for depressive symptoms, was asked of each household’s respondent. Although not specifically validated for the Myanmar context, it has been used previously in this region and in epidemiological studies to assess depressive symptoms across the world [19, 20]. Mid-upper arm circumference ( MUAC) data for children ages 6 to 59 months were used to categorize children with mild (12.5 to < 13.5 cm), moderate (11.5 to < 12.5 cm), and severe (< 11.5 cm) acute malnutrition. MUACs less than 22.5 cm were considered malnourished among women of reproductive age.

Mortality rates for infants less than 1 year of age (IMR) and for children under 5 years old (U5MR) were calculated as a ratio of deaths per thousand live births using standard approaches. Crude mortality and age-specific death rates (ASDR) were calculated using a ratio of deaths to mid-year population. Mortality rate ratios were estimated using Poisson regression with an offset for the number of household members at-risk for outcome events within relevant age groups.

Additional health outcomes measured for WRA included unmet need for contraception and who attended their last delivery. Additional health outcomes among children included presence of diarrhea in the 2 weeks prior to the survey and receipt of deworming and Vitamin A among household children. Prevalence of *Plasmodium falciparum* malaria was measured by sampling all household members of the first, 15th, and 30th household in each cluster. In villages with less than 30 households a household was chosen at random such that 3 households per village were completely sampled.

### Human rights violations

Respondents were asked to report household exposure to human rights violations (HRVs) within the year prior to the survey, using a module previously developed for use in the region [20, 21]. HRVs included forced labor, destruction/seizure of food, livestock, or crops, confiscation of land, physical injuries, detention, and landmine injuries.

### Analysis

The primary independent variable was migration status. For household-level associations we assigned households to three mutually exclusive migration categories based on the reported presence of at least one in-migrant or out-migrant. Households with neither in- nor out-migrants were categorized as “non-migrant” households; and we excluded from analyses 24 households that included both in-migrants (*n* = 92) and out- migrants (*n* = 102).

In order to explore possible differences between internal (within Myanmar) and international migrants, we formally compared, separately for in-migrants and out-migrants, the demographic composition (age and sex) and stated reasons for migration between internal and international migrants.

Frequencies and the weighted means and proportions of demographic characteristics for individuals and households were tabulated for the three migration categories, and three pairwise comparisons were made using t-test and adjusted Wald test as appropriate [22, 23].

Health and human rights outcomes were tabulated by household migration status, and crude and adjusted risk ratios were estimated using generalized linear models with a log link function, using non-migrant households as the comparator group. Adjusted mortality rate ratios were calculated using poisson regression with an offset based on the number of eligible individuals (i.e. children under 5) in the household. Adjusted models accounted for household size, educational attainment, marital status of the respondent (head of household) and stratum (CBO target population). Factors used to adjust these models were based on empiric and theoretical work done previously in this region [11–18].

We calculated sampling weights as the inverse sampling probability of being in a selected household; and accounted for design effects due to clustering at the village level. All analyses were conducted in R statistical software [24] using the “survey” package [25] to account for the multi-staged, stratified design of the cluster survey.

### Sample size

Sample size was based on a balance between continued monitoring of the U5MR with reasonable precision within each CBO service area (stratum) and operational feasibility under security and resource constraints. The sample size of 1350 households in each stratum allowed for precision of U5MR for each CBO service area to within 50/1000 live births, [18]. Security concerns led to the replacement of 10 out of 225 planned clusters (6750 households), and prevented sampling in 6 clusters. The final sample included 219 clusters, 29 of which had fewer than 30 households, and a total of 30,323 people in 6, 178 households. The response rate was 91.5%. Data available for the present analysis provided over 90% power to detect 10 percentage point differences in reasons for migration between in-migrants (*n* = 240) and out-migrants (*N* = 1534); and over 80% power to detect a 75% relative increase in the proportion of out-migrant households (*n* = 1010) experiencing key health-related outcomes of depressive symptoms and under five global acute malnutrition, compared to non-migrant households (*n* = 5080). Power was substantially lower (~ 40–50%) to detect differences in health outcomes among in-migrant households (*n* = 110); or differences in risk of crude or child mortality.

### Ethical approval

Each head of household provided informed verbal consent. Surveyors referred cases of malaria, malnutrition, or emotional distress to local community leaders to facilitate appropriate care, via well-established networks of clinics and community health workers. Due to ethical implications of violating anonymity, no formal evaluation of these referral mechanisms was done. Heads of household 15 or older were interviewed. According to the Demographic and Health Survey 2015–16, 19% of women and 7% of men between the ages of 25–34 were married by the age of 18 [26]. Thus, as heads of household in Eastern Burma may be as young as 15, additional consent from a guardian was not sought for participants who were 15–18 years old and living independently of their parents. This is in accordance with guidance provided by the organization Ethical Research Involving Children [27]. The Institutional Review Boards at the University of California Los Angeles and Partners HealthCare provided ethical review and approved the study protocol, including the verbal consent process.

## Results

### Migrant demographics

**Fig. 1:**
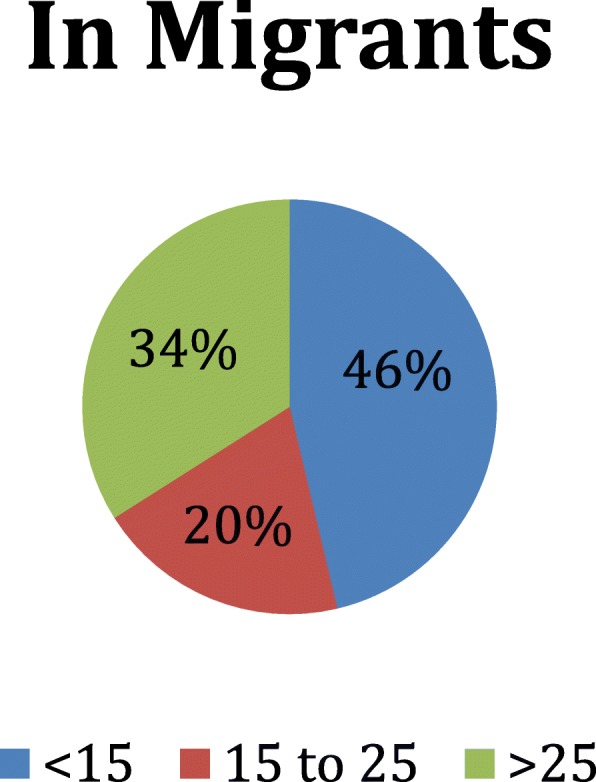
Age Distribution of In-Migrants

**Fig. 2:**
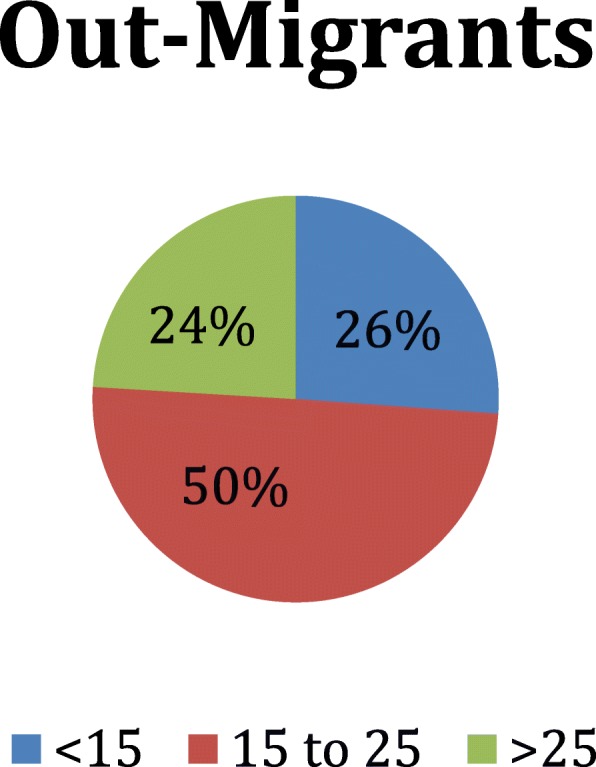
Age Distribution of Out-Migrants

Demographic composition of the overall sample has been previously reported [18]. Table 1 presents data on individuals who migrated into or out of households in the surveyed region in the previous 12 months. Individuals who migrated out of households more than 12 months prior to the survey were classified as non-migrants. Of the 31,851 individuals included in this sample, only 240 migrated into the household in the previous year; more than six times as many individuals had migrated out of the household (*n* = 1534) (Table 1). Those who migrated out of eastern Myanmar were more likely to be male (55.2%), and three times more likely to be between the ages of 15–25 (49.5%) than non-migrants. Those migrating out of Myanmar were most likely to be children of the survey respondent (79.5%; Table 1). The age distribution of those migrating in and out of households are show in Figs. 1 and 2. Tables 2 and 3 present data on households that included one or more members who had migrated in, out, or had no migration in the 12 months prior to the survey. Household-level results exclude the 24 households with both in- and out-migrants present. Of note, the primary respondent reported the reasons for migration, not the migrant themselves.

**Table 1:** Age, Gender, Relationship of Migrant to Respondent, Site of and Reason For Migration

	In-migrants	Out-migrants	Non-migrants	Significance
Households	112	1010	5080	
Persons	240	1534	30,077	
Age	Mean (95% CI)	Mean (95% CI)	Mean (95% CI)	A B
	20.8 (17.1–24.5)	20.3 (19.4–21.2)	24.9 (24.3–25.5)	
	n, % (95% CI)	n, % (95% CI)	n, % (95% CI)	B C
< 15	99, 46.3% (34.7–57.8%)	338, 26.0% (20.1–31.8%)	11,600, 40.1% (38.8–41.4%)	
15 to 25	55, 19.7% (12.8–26.6%)	774, 49.5% (43.8–55.1%)	5127, 16.3% (15.5–17.1%)	
> 25	86, 34.0% (26.6–41.5%)	413, 23.9% (21.0–26.8%)	13,259, 43.2% (42.0–44.5%)	
Sex	n, % (95% CI)	n, % (95% CI)	n, % (95% CI)	B
Male	120, 48.2% (42.7–53.6%)	860, 55.2% (50.4–59.9%)	14,967, 49.7% (48.9–50.4%)	
Female	120, 51.8% (46.4–57.3%)	656, 44.8% (40.1–49.6%)	15,104, 50.3% (49.6–51.1%)	
Relationship to Respondent	n, % (95% CI)	n, % (95% CI)	n, % (95% CI)	A B C
Self	32, 14.2% (11.0–17.4%)	0, 0.0% (0.0–0.0%)	6146, 20.3% (19.7–21.0%)	
Parent	5, 1.8% (0.0–4.0%)	20, 0.9% (0.3–1.6%)	1153, 3.6% (3.2–4.1%)	
Spouse	19, 8.3% (5.6–11.1%)	78, 4.7% (3.3–6.0%)	4915, 16.3% (15.8–16.8%)	
Child	94, 45.5% (32.6–58.3%)	1135, 79.5% (75.7–83.4%)	14,735, 50.3% (48.7–51.9%)	
Uncle/Aunt	2, 0.7% (0.0–1.7%)	05, .5% (0.0–1.1%)	81, 0.3% (0.2–0.4%)	
Sibling	8, 1.4% (0.2–2.6%)	106, 4.6% (2.8–6.5%)	706, 2.0% (1.5–2.5%)	
Niece/Nephew	26, 9.3% (3.3–15.4%)	38, 2.6% (1.3–3.9%)	593, 2.5% (1.8–3.2%)	
Friend / other	54, 18.8% (8.4–29.2%)	152, 7.1% (4.9–9.3%)	1733, 4.7% (3.9–5.5%)	
Where Did They Move	n, % (95% CI)	n, % (95% CI)		C
In State	117, 71.7% (53.5–89.9%)	508, 32.4% (26.5–38.2%)		
In Burma	51, 17.0% (5.0–28.9%)	235, 17.1% (10.9–23.3%)		
Thailand	27, 11.3% (1.9–20.7%)	746, 48.2% (40.9–55.5%)		
Malaysia	0, 0.0% (0.0–0.0%)	24, 1.2% (0.3–2.0%)		
Other	0, 0.0% (0.0–0.0%)	13, 1.2% (0.0–2.3%)		
Primary reason for moving	n, % (95% CI)	n, % (95% CI)		C
Work	54, 23.2% (7.9–38.5%)	744, 40.2% (31.4–49.0%)		
Education	31, 14.2% (2.5–25.9%)	562, 46.4% (39.8–53.0%)		
Family	39, 26.3% (12.3–40.3%)	52, 2.7% (1.6–3.9%)		
Marriage	28, 16.1% (0.8–31.4%)	84, 6.6% (4.5–8.6%)		
Insecurity	7, 3.2% (0.0–9.1%)	10, 1.3% (0.0–2.9%)		
Improved Security	14, 7.0% (0.0–15.4)	1, 0.1% (0.0–0.4%)		
Land Confiscated	37, 4.5% (1.3–7.8%)	2, 0.2% (0.0–0.6%)		
No Reason/Other	13, 5.5% (1.0–10.0%)	43, 2.5% (1.4–3.5%)		

“A” *p* < 0.05 for test of no difference between IN-migrants vs. NON-migrants

“B” *p* < 0.05 for test of no difference between OUT-migrants vs. NON-migrants

“C” *p* < 0.05 for test of no difference between IN-migrants vs. OUT-migrants

Analysis done with regard to individuals, thus 24 HH with in and out migration added to in and out columns, respectively, twice, in order to facilitate analysis of individual characteristics

**Table 2:** Mean Number of Migrants by Type, per Household

	HH with in-migration	HH with out-migration	HH with no migration	Significance
	Mean or % (95% CI)	Mean or % (95% CI)	Mean or % 95% CI)	
In-migrants per HH	2.4 (1.8, 3)	0.0 (0, 0)	0.0 (0, 0)	
Out-migrants per HH	0.0 (0, 0)	1.5 (1.4, 1.7)	0.0 (0, 0)	
Non-migrants per HH	3.3 (2.4, 4.1)	5.0 (4.7, 5.4)	4.9 (4.7, 5.1)	A C
Current residents	5.6 (5.1, 6.2)	5.0 (4.7, 5.4)	4.9 (4.7, 5.1)	A C
Total HH size	5.6 (5.1, 6.2)	6.6 (6.3, 6.9)	4.9 (4.7, 5.1)	A B C
Male proportion	49% (44–53%)	^a^50% (49-52%)	49% (48–50%)	

“A” *p* < 0.05 for test of no difference between IN-migrants vs. NON-migrants

“B” *p* < 0.05 for test of no difference between OUT-migrants vs. NON-migrants

“C” *p* < 0.05 for test of no difference between IN-migrants vs. OUT-migrants

24 HH with in and out-migration omitted from this analysis

**Table 3:** Composition of Household, Characteristics of Household Respondent

Composition of HH	n, mean or % (95% CI)	n, mean or % (95% CI)	n, mean or % (95% CI)	Significance
# nuclear family members	370, 4.3 (3.9–4.7)	5330, 5.6 (5.3–5.9)	21,688, 4.3 (4.2–4.5)	
Spouse present	71, 81.3% (73.5–89.1%)	781, 80.5% (78–83%)	4114, 81.1% (78.9–83.2%)	
# of dependent children	154, 1.9 (1.5–2.4)	2251, 2.5 (2.3–2.7)	9665, 2.0 (1.9–2.1)	B C
# extended family members	132, 1.3 (0.9–1.8)	1079, 0.9 (0.8–1.1)	3051, 0.6 (0.5–0.6)	A B
# grandparents	17, 0.1 (0–0.3)	161, 0.2 (0.1–0.2)	678, 0.1 (0.1–0.2)	
# parents of adult without children	3, 0.0 (0.0–0.1)	54, 0.0 (0–0.1)	196, 0.0 (0–0)	
# niece/nephew, friend/other	1.0 (0.6, 1.5)	0.6 (0.5, 0.7)	0.3 (0.3, 0.4)	A B
Single parent family	13, 14.3% (7.2–21.4%)	191, 18.8% (16 21.6%)	599, 11.9% (10.6–13.1%)	B
Single parent family with children < 5	6, 7.7% (1.1–14.3%)	47, 4.2% (2.7–5.7%)	123, 2.5% (2–3.1%)	B
Three or more generation HH	15, 13.8% (5.1–22.5%)	143, 14.0% (10.9–17.2%)	564, 11.5% (10–12.9%)	
Characteristics of household respondents^a^	n, % (95% CI)	n, % (95% CI)	n, % (95% CI)	
Educational attainment				B
None	27, 30.8% (18.3–43.2%)	427, 45.3% (38.7–51.9%)	2054, 41.9% (37.7–46%)	
1 to 5 standard	33, 36.0% (24.7–47.2%)	358, 36.5% (30.9–42.2%)	1833, 37.2% (33.6–40.9%)	
6 to 10 standard	17, 24.1% (7.8–40.4%)	109, 10.9% (8.2–13.6%)	735, 13.5% (11.4–15.5%)	
Above 10 standard	2, 3.2% (0–8.4%)	10, 0.8% (0.2–1.4%)	90, 1.6% (1–2.2%)	
Other education	8, 5.2% (1.2–9.2%)	80, 6.4% (4.1–8.6%)	329, 4.9% (3.6–6.1%)	
Don’t know / refused	1, 0.7% (0–2.1%)	2, 0.1% (0–0.3%)	35, 0.9% (0.4–1.5%)	
Language				A C
Pwo Karen	9, 9.9% (1.1–18.6%)	108, 11.1% (4.6–17.7%)	530, 11.9% (7.1–16.7%)	
Sgaw Karen	53, 74.4% (60.4–88.4%)	390, 53.0% (42.8–63.2%)	2210, 54.8% (47.5–62%)	
Burmese	14, 11.1% (2.3–19.9%)	130, 11.9% (6.8–16.9%)	671, 11.9% (7.4–16.4%)	
Shan	0, 0.0% (0–0%)	43, 7.3% (1.6–13.1%)	129, 4.2% (0.9–7.6%)	
Karenni	0, 0.0% (0–0%)	124, 6.6% (4.1–9.2%)	515, 6.1% (4.5–7.7%)	
Mon	12, 4.6% (1.6–7.6%)	171, 7.4% (4.4–10.2%)	946, 9.0% (5.4–12.6%)	
Other	0, 0.0 (0–0%)	20, 2.6% (0.4–4.9%)	75, 2.1% (0.3–3.9%)	
Ethnicity				A C
Karen	65, 86.5% (78.1–94.9%)	505, 64.6% (55.3–73.8)	2740, 66.8% (60.4–73.2%)	
Karenni	1, 0.9% (0–2.6%)	144, 8.9% (5.4–12.4%)	768, 11.2% (7.4–14.9%)	
Shan	1, 0.7% (0–2.2%)	29, 4.6% (0.3–9%)	91, 3.2% (0.2–6.3%)	
Mon	18, 9.8% (2.9–16.7%)	224, 10.4% (5.6–15.2%)	1157, 10.8% (7.1–14.4%)	
Burmese	2, 1.5% (0–3.4%)	015, .9% (0.1–1.6%)	63, 0.8% (0.4–1.2%)	
Other	1, 0.7% (0–2%)	69, 10.6% (3.2–18%)	257, 7.2% (2.9–11.6%)	
Religious affiliation				
Christian	19, 24.6% (14.1–35.1%)	267, 35.5% (25.5–45.4%)	1522, 34.9% (28–41.7%)	
Buddhist	61, 61.6% (44–79.1%)	647, 57.1% (46.9–67.3%)	3260, 59.0% (52–65.9%)	
Muslim	0, 0.0% (0–0%)	1, 0.1% (0–0.3%)	6, 0.1% (0–0.3%)	
Animist	8, 13.8% (0–28%)	67, 6.8% (2.6–11%)	257, 5.4% (3.2–7.6%)	
None	0, 0.0% (0–0%)	0, 0.0 (0–0%)	4, 0.1% (0–0.2%)	
Other	0, 0.0% (0–0%)	4, 0.5% (0–1.1%)	27, 0.5% (0–1%)	

“A” *p* < 0.05 for test of no difference between IN-migrants vs. NON-migrants

“B” *p* < 0.05 for test of no difference between OUT-migrants vs. NON-migrants

“C” *p* < 0.05 for test of no difference between IN-migrants vs. OUT-migrants

24 HH with in and out-migration omitted from this analysis

### Origin and destination of migration

#### In-migrants

**Fig. 3:**
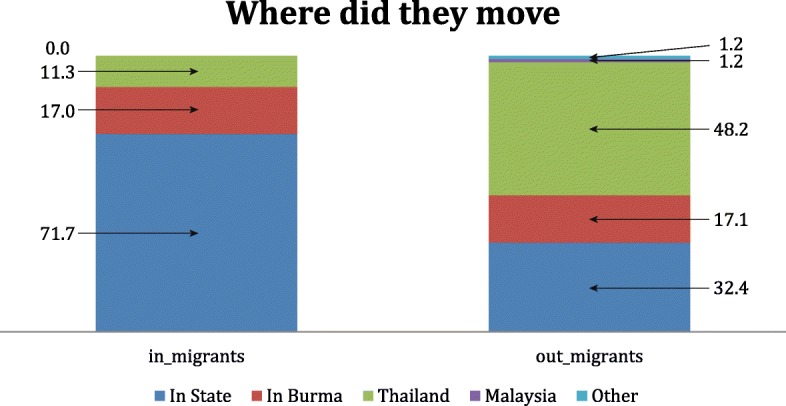
Where migrants moved

**Fig. 4:**
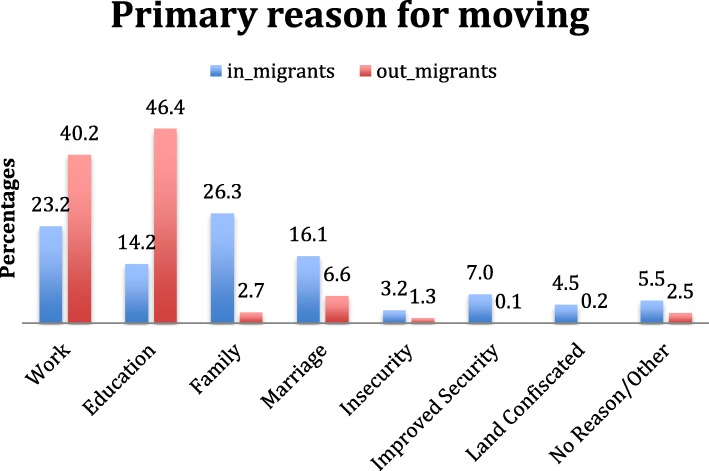
Primary Reason for Moving

Most in-migrants had moved to their present location in the study area from other areas in Myanmar (87%), with a majority (71.7%) moving from within the same state (Table 1 and Fig. 3). Only 11.3% (27 individuals) had returned from another country (Thailand). The primary reasons cited for a return to the household were family or marriage (42.4%), followed by work (23.2%) and education (14.2%); security (10.3%) or land confiscation (4.5%) was cited by 14.7% of in-migrants (Fig. 4).

#### Out-migrants

Out-migrants tended to move farther from home, with over two-thirds moving outside the State: 17.1% moved to another state within Myanmar; and half of out-migrants had departed for Thailand (48.2%), Malaysia (1.2%), or other countries (1.2%). Coupled with the six-fold larger size of the out- migrant population, the absolute number of international migrants leaving Myanmar (*n* = 783) was 29 times larger than the number returning from abroad (*n* = 27). Education (46.4%) and work (40.2%) were cited by 86.6% respondents as the primary reasons household members had left; nearly twice the proportion citing such reasons for migration into households in the survey area (49.5%). In contrast, family/marriage (9.3% vs. 39.4%), and security/land confiscation (1.6% vs. 14.7%) were less often cited among out-migrant households (Fig. 4).

Households with out-migration tended to be larger than those with in- or no migration, with an average household size of 6.6 (vs. 5.6 with in-migration, 5.0 with no migration). Mean number of individuals migrating out of a household was 1.5 (Table 2).

### Family composition and migration

Migrants tended to come into a household in groups of 2 or more (mean 2.4, Table 2); and were more likely to move out alone or in pairs (mean 1.5. Family composition differed between households with in-, out-, or no migration. For example, respondents from households with migrants returning into the home tended to have respondents that speak Sgaw Karen, and were more likely to be of Karen ethnicity, than household respondents with no migration or out-migration. Households with out-migration had a higher proportion of dependent children than households with either no- or in-migration: 2.5 vs 2.0 or 1.9, respectively. Households with in- or out-migration had a larger number of extended family members, and similarly had a higher number of nieces, nephews, or friends/other. A significantly higher number of households with out-migration were single parent households, and single parent households with children under 5, than households with no migration. Respondents queried in households with outward migration tended to have lower overall education levels than household respondents without migration (Table 3). The religious affiliations of household respondents within all migration subgroups were similar (Table 3).

### Reasons for migration

#### In-migration

Table 4 presents reasons for in- and out-migration, stratified by age and sex. Persons migrating into households in Eastern Myanmar did so for a variety of reasons. A substantial proportion of in-migrants did so for “family” reasons (38.1% of men under 15, 38.1% of women under 15, and 32.3% of women over 25). Substantial proportions of all age groups migrated back for “marriage.” Thirty-two percent of men age 15–25, and 44.8% of men over 25 migrated back to their households for work; 23.2% of women under 15 and 28.2% of women between 15 and 25 returned to their households for an education.

**Table 4:** Primary Reason for Moving

	Men			Women		
In-migrants						
Primary reason for moving	Age < 15 *N* = 48 % (95% CI)	15–25 *N* = 22 % (95% CI)	> 25 *N* = 50 % (95% CI)	Age < 15 *N* = 51 % (95% CI)	15–25 *N* = 33 % (95% CI)	> 25 *N* = 36 % (95% CI)
Work	21.0% (0–46%)	32.3%(7.1–57.6%)	44.8% (24.7–64.9%)	3.3% (0–8.8%)	18.5% (3.5–33.6%)	30.4% (8.1–52.7%)
Education	15.0% (0.2–29.9%)	20.0% (0–42.6%)	0.0% (0–0%)	23.2% (0–47%)	28.2% (0–57%)	1.5% (0–4.5%)
Family	38.1% (17.9–58.4%)	10.7% (0–30.2%)	13.1% (2.6–23.6%)	38.1% (21.4–54.9%)	6.1% (0–17.7%)	32.35% (12.2–52.3%)
Marriage	15.15% (0–35.8%)	18.2% (0–40.7%)	12.2% (0–28%)	16.5% (0–40.8%)	28.5% (8.1–48.9%)	10.3% (0–26.9%)
Insecurity	2.4% (0-%7.2)	0.0% (0–0%)	2.6% (0–7.8%)	2.1% (0–6.3%)	5.6% (0–13.8%)	6.4% (0%18.8)
Improved Security	6.8% (0–20.3%)	0.0% (0–0%)	7.6% (0–18.4%)	11.3% (0–26.7%)	3.6% (0–10.8%)	6.2% (0–14.9%)
Land Confiscated	0.6% (0–1.8%)	9.8% (0.3–19.3%)	6.5% (1.4–11.5%)	3.5% (0–7.7%)	5.8% (0.8–10.8%)	5.5% (0.4–10.6%)
No Reason/Other	1.1% (0–3.4%)	9.0% (0,-20.5%)	13.2% (1.1–25.3%)	2.0% (0–5%)	3.6% (0–10.8%)	7.4% (0–16.5%)
Out-migrants						
Primary reason for moving	Age < 15 *N* = 169 % (95% CI)	15–25 *N* = 426 % (95% CI)	> 25 *N* = 260 % (95% CI)	Age < 15 *N* = 168 % (95% CI)	15–25 *N* = 333 % (95% CI)	> 25 *N* = 151 % (95% CI)
Work	8.5% (3.1–13.8%)	54.5% (45.5–63.6%)	66.7% (55–78.4%)	5.2% (0.9–9.6%)	38.2% (28.6–47.7%)	55.9% (37.6–74.1%)
Education	83.0% (76.5–89.5%)	40.4% (31.7–49.1%)	7.5% (2.4–12.7%)	89.1% (83.8–94.5%)	49.7% (40.4–59%)	6.8% (0.3–13.3%)
Family	6.2% (1.6–10.9%)	0.5% (0.1–1%)	2.0% (0.6–3.5%)	3.4% (1.2–5.6%)	2.1% (0–4.3%)	5.7% (0.9–10.4%)
Marriage	0.0% (0–0%)	2.0% (0.4–3.7%)	17.8% (7.3–28.4%)	0.5% (0–1.3%)	7.3% (3.5–11.2%)	16.4% (7.8–25%)
Insecurity	0.0% (0–0%)	0.5% (0–1.4%)	1.0% (0–2.3%)	0.0% (0–0%)	0.8% (0–2.3%)	8.1% (0–22.3%)
Improved Security	1.1% (0–3.2%)	0.0% (0–0%)	0.0% (0–0%)	0.0% (0–0%)	0.0% (0–0%)	0.0% (0–0%)
Land Confiscated	0.0% (0–0%)	0.5% (0–1.3%)	0.8% (0–2.4%)	0.0% (0–0%)	0.0% (0–0%)	0.0% (0–0%)
No Reason/Other	1.3% (0–2.6%)	1.6% (0.1–3%)	4.1% (1–7.1%)	1.7% (0–4%)	1.9% (0–3.8%)	7.2 (2.2–12.3%)

24 HH with in and out-migration omitted from this analysis

#### Out-migration

Patterns of out-migration differed by gender. Although both male and female out-migrants under the age of 15 left primarily to seek an education (83.0, 89.1%), women aged 15–25 left primarily to seek an education(49.7%), whereas a majority of women over the age of 25 migrated out of the household to seek employment (55.9%). In contrast, men over the age of 15 left the household primarily in order to seek work (age 15–25, 32.3%; age > 25, 44.8%).

We formally compared and found no significant differences between the demographic composition and stated reasons for migration between internal and international migrants. We therefore proceeded to analyze internal and international migrants together in the two originally proposed categories (in- and out-migrant groups).

### Migration and health outcomes

#### In-migration

Table 5 presents adjusted associations between household migration and health outcomes, as well as access to health care using prevalence ratios. Compared to households with no migration, households with in-migration were less likely to own a latrine (adjusted PR 0.60; 95% CI 0.38, 0.94); women of reproductive age living in these households were more likely to be acutely malnourished (mid-upper arm circumference less than 22.5; aPR 2.08; 95% CI 1.29, 3.36). However, global acute malnutrition was less common among children under 5 living in in-migrant vs. non-migrant households (0.5% vs. 8.7%, aPR 0.06; 95% CI 0.01, 0.49). Households with in-migration had a higher proportion of unmet need for contraception (aPR 1.58; 95% CI 1.11, 2.26), defined as the proportion of women not currently using contraceptive methods among those who stated they were not planning to have more children, or who were not currently pregnant and did not explicitly state that they did not need contraception [18]. No clear patterns emerged with regards to skilled attendance at birth.

**Table 5:** Associations between Health Outcomes and Migration

	HH with in-migrants		HH with out-migrants		HH without migrants		Adjusted Risk Ratio: IN vs. NON (95% CI)	Adjusted Risk Ratio: OUT vs. NON (95% CI)
Respondents	n, % (95% CI)		n, % (95% CI)		n, % (95% CI)			
PHQ Depression Scale								
0–3	89, 99.1% (97.4–100%)		901, 91.7% (87.9–95.4%)		4764, 95.6% (94.4–96.8%)		1.03 [1.00, 1.05]	0.96 [0.92, 1.00]
4–6	1, 0.9% (0–2.6%)		78, 8.3% (4.6–12.1%)		252, 4.4% (3.2–5.6%)		0.25 [0.03, 1.88]	1.85 [1.16, 2.97]
Self-Reported Health								
Good	42, 43.1% (27.8–58.3%)		473, 38.6% (32.4–44.8%)		2605, 45.5% (40.7–50.2%)		0.94 [0.65, 1.37]	0.97 [0.84, 1.11]
Fair	37, 46.7% (32.2–61.2%)		412, 48.7% (43.8–54.6%)		2016, 45.8% (41.2–50.4%)		1.05 [0.77, 1.43]	0.97 [0.88, 1.08]
Poor	12, 10.2% (2.3–18.2%)		105, 12.7% (8.4–17%)		433, 8.7% (7.1–10.4%)		1.17 [0.57, 2.40]	1.28 [0.90, 1.81]
Health Care Provider	n, % (95% CI)		n, % (95% CI)		n, % (95% CI)		Adjusted Risk Ratio: IN vs. NON (95% CI)	Adjusted Risk Ratio: OUT vs. NON (95% CI)
Government Health Center	6, 6.7% (0–15.7%)		60, 8.1% (4.2–12.1%)		383, 8.3% (6.1–10.6%)		0.84 [0.28, 2.56]	1.24 [0.76, 2.01]
Ethnic Clinic	20, 31.6% (15.5–47.8%)		113, 18.4% (11.3–25.6%)		612, 18.2% (13.2–23.3%)		1.40 [0.87, 2.24]	0.93 [0.70, 1.25]
VHW or Medic	26, 41.6% (24.3–58.9%)		332, 45.5% (34.9–56.2%)		1673, 51.2% (44.1–58.3%)		0.78 [0.48, 1.25]	0.90 [0.73, 1.12]
Traditional Healer	2, 5.8% (0–12.9%)		8, 1.3% (0.2–2.4%)		63, 2.6% (1.3–3.8%)		4.16 [1.33, 13.02]	0.44 [0.16, 1.17]
Friend or Relative or Family	0, 0.0% (0–0%)		41, 7.7% (2.2–13.2%)		159, 4.5% (2.9–6.2%)		0.00 [0.00, 0.00]	1.53 [0.89, 2.62]
Pharmacy	7, 14.2% (3.2–25.3%)		93, 18.9% (13–24.8%)		383, 15.1% (10.2–20.1%)		0.87 [0.43, 1.75]	1.18 [0.93, 1.50]
All subjects	n, % (95% CI)		n, % (95% CI)		n, % (95% CI)	Adjusted Risk Ratio: IN vs. NON (95% CI)		Adjusted Risk Ratio: OUT vs. NON (95% CI)
Slept under ITN last night	393, 72.5% (58.4–86.7%)		3277, 64.5% (58.1–70.9%)		16,725, 63.5% (58.2–68.7%)		1.08 [0.89, 1.30]	1.08 [0.99, 1.17]
Pf positive (RDT)	2, 3.8% (0–10.8%)		10, 2.5% (0.3–4.6%)		33, 2.1% (0.9–3.2%)		0.97 [0.10, 9.05]	1.08 [0.38, 3.09]
WRA	n, % (95% CI)		n, % (95% CI)		n, % (95% CI)	Adjusted Risk Ratio: IN vs. NON (95% CI)		Adjusted Risk Ratio: OUT vs. NON (95% CI)
Malnutrition among WRA	13, 17.8% (6–29.6%)		99, 12.9% (9.2–16.7%)		383, 10.7% (8.4–13.1%)		2.08 [1.29, 3.36]	1.09 [0.78, 1.52]
Unmet Need for Contraception	18, 34.5% (21.9–47.1%)		119, 32.7% (25.8–39.6%)		454, 19.7% (16.9–22.5%)		1.58 [1.11, 2.26]	1.22 [0.95, 1.56]
Who attended last delivery?	n, % (95% CI)		n, % (95% CI)		n, % (95% CI)	Adjusted Risk Ratio: IN vs. NON (95% CI)		Adjusted Risk Ratio: OUT vs. NON (95% CI)
Doctor	6, 6.7% (0.5–12.9%)		21, 6.4% (2.3–10.4%)		160, 5.0% (3.4–6.5%)		1.49 [0.62, 3.59]	1.32 [0.62, 2.82]
HA	6, 9.6% (0.8–18.3%)		19, 5.95 (2.1–9.7%)		110, 6.1% (3.3–8.8%)		1.95 [0.84, 4.53]	1.38 [0.86, 2.22]
Medic	15, 23.7% (6.8–40.6%)		39, 9.9% (5.1–14.7%)		187, 6.8% (4.3–9.3%)		1.70 [1.00, 2.91]	1.36 [0.90, 2.06]
TBA	21, 52.0% (27.7–76.3%)		22, 64.5% (56–72.9%)		1406, 74.2% (68.9–79.5%)		0.80 [0.54, 1.18]	0.88 [0.78, 0.99]
Other	5, 8.0% (0.6–15.4%)		34, 13.4% (6.5–20.2%)		102, 8.0% (4.6–11.3%)		0.99 [0.35, 2.86]	1.23 [0.78, 1.94]
Doctor (binary version)	6, 6.1% (0.4–11.8%)		246.2 (2.5–10%)		195, 5.4% (3.8–7%)		1.24 [0.50, 3.03]	1.24 [0.62, 2.49]
HA (binary version)	8, 10.0% (1.6–18.4%)		28, 6.6% (3.1–10.2%)		144, 6.9% (3.9–9.9%)		1.79 [0.83, 3.83]	1.38 [0.88, 2.15]
Medic (binary version)	26, 24.2% (8.4–39.9%)		60, 15.9% (10.2–21.7%)		292, 10.3% (7.3–13.3%)		1.50 [0.91, 2.48]	1.45 [1.04, 2.02]
THW (binary version)	24, 54.8% (32–6,77%)		253, 68.6% (60.6–76.5%)		1635, 77.1% (72.-81.7%)		0.80 [0.57, 1.13]	0.89 [0.80, 0.99]
Other (binary version)	9, 13.4% (4.1–22.6%)		41, 15.3% (8.5–22.1%)		194, 14.1% (9–19.2%)		0.97 [0.45,2.09]	0.87 [0.56,1.35]
Children under 5	n, % (95% CI)		n, % (95% CI)		n, % (95% CI)	Adjusted Risk Ratio: IN vs. NON (95% CI)		Adjusted Risk Ratio: OUT vs. NON (95% CI)
Slept under ITN last night	69, 80.7% (68.6–92.8%)		420, 72.6% (64.8–80.5%)		2477, 68.7% (62.7–74.7%)		1.10 [0.96, 1.27]	1.13 [1.02, 1.24]
MUAC								
Mild	3, 4.2% (0–10%)		50, 15.2% (11.3–19.1%)		334, 14.4% (11.7–17%)		0.33 [0.08,1.36]	1.09 [0.78,1.52]
Moderate	1, 0.7% (0–2.1%)		24, 6.6% (3.7–9.5%)		142, 6.3% (4.4–8.3%)		0.13 [0.02, 0.99]	1.24 [0.73, 2.12]
Severe	0, 0.0% (0–0%)		13, 4.0% (1.6–6.5%)		97, 5.4% (2.9–7.9%)		0.00 [0.00, 0.00]	0.75 [0.40, 1.38]
GAM (Moderate or severe)	1, 0.5% (0–1.4%)		37, 7.3% (4.3–10.2%)		239, 8.7% (5.9–11.4%)		0.06 [0.01, 0.49]	0.95 [0.59, 1.51]
Child Diarrhea, 2wks	18, 26.2% (13.9–38.6%)		127, 24.6% (18.6–30.5%)		617, 18.4% (15.7–21%)		1.36 [0.86, 2.15]	1.34 [1.03, 1.73]
Receipt of Vitamin A	14, 28.8% (9.7–47.9%)		182, 42.2% (34.2–50.3%)		1107,40.8% (34.9–46.8%)		0.82 [0.49, 1.37]	1.07 [0.88, 1.30]
Receipt of Deworming	27, 48.1% (30.8–65.4%)		206, 53.8% (45.8–61.9%)		1345, 55.4% (49.9–61%)		0.87 [0.58, 1.31]	0.98 [0.84, 1.14]
Birth record	n, % (95% CI)		n, % (95% CI)		n, % (95% CI)	Adjusted Risk Ratio: IN vs. NON (95% CI)		Adjusted Risk Ratio: OUT vs. NON (95% CI)
No record	40, 47.4% (35.8–58.9%)		284, 48.8% (40.7–56.9%)		1853, 56.4% (50.6–62.2%)		0.83 [0.63,1.09]	0.85 [0.76,0.96]
Ethnic record	23, 33.6% (20–47.1%)		139, 36.6% (27.9–45.3%)		783, 32.2% (26–38.3%)		1.07 [0.74, 1.53]	1.12 [0.92, 1.37]
Government record	7, 10.3% (0.8–19.8%)		94, 12.7% (6.8–18.5%)		511, 9.9% (7.3–12.5%)		1.22 [0.50, 2.98]	1.76 [1.20, 2.59]
Other country’s record	9, 8.7% (1.3–16.1%)		21, 2.0% (0.7–3.2%)		95, 1.5% (0.9–2.2%)		2.31 [0.83, 6.47]	1.17 [0.72, 1.91]
HH level outcomes	n, % or Mean (95% CI)		n, % or Mean (95% CI)		n, % or Mean (95% CI)	Adjusted Risk Ratio: IN vs. NON (95% CI)		Adjusted Risk Ratio: OUT vs. NON (95% CI)
Household has latrine	44, 31.0% 18.2–43.8%)		606, 51.9% (43.5–60.3%)		3075, 53.4% (47.8–59.1%)		0.60 [0.38, 0.94]	0.94 [0.83, 1.08]
Number of ITNs per person	0.42 (0.35, 0.49)		0.46 (0.40,0.53)		0.41 (0.38, 0.45)		0.02 [−0.05, 0.09]	0.14 [0.08, 0.19]
Mortality	Deaths	Incidence per 1000	Deaths	Incidence per 1000	Deaths	Incidence per 1000	Adjusted Incidence	Adjusted Incidence
Crude mortality rate	2	6 (0, 15.3)	51	9 (4.6, 12.8)	177	9 (6.5, 11.7)	0.72 [0.14, 3.60]	0.94 [0.57, 1.57]
Child death ASDR-5	2	40 (0, 100)	16	42(13.4, 71)	65	28 (17.4,39.2)	1.62 [0.30, 8.82]	1.53 [0.71, 3.30]

24 HH with in and out-migration omitted from this analysis

Of note, some individual health outcomes among in-migrants differed significantly when compared to non-migrants. Compared to people who did not move, in-migrants were more likely to be positive for *P. falciparum* malaria (15.3% vs. 2.1%, aPR 5.02, 95% CI 1.56, 16.15), though they were more likely to have slept under an ITN the night before (89.1% vs. 69.1%, aPR 1.23, 95% CI 1.01, 1.50). In-migrant children were less likely to have received Vitamin A supplementation than non-migrant children (0.30, 95% CI 0.10, 0.91).

#### Out-migration

Respondents from households that reported any out-migration during the past year were more likely to screen positive for depressive symptoms than respondents from households with no migration (aPR 1.85; 95% CI 1.16, 2.97). Individuals in households with out-migration had greater access to insecticide treated nets (0.14 more ITNs per person, *p* < 0.05) and were more likely to have slept under one on the night prior to the survey than individuals in households with no migration (aPR 1.13; 95% CI 1.02, 1.24). Children in households with out-migration were more likely to have had diarrhea in the past 2 weeks than households with no migration (aPR 1.34; 95%CI 1.03, 1.73). They also were more likely to have a government birth certificate than children households with no migration (aPR 1.76; 95% CI 1.20, 2.59).

### Migration and human rights violations

The distribution of human rights violations in this population has been previously described. [18] Nearly one in ten households in eastern Myanmar suffered at least one human rights violation in the 12 months prior to the survey (10.7%; 95% CI 7.0–14.5) [18] including 10.3% of households with in-migration and 7.8% of households with out-migration. The most common human rights violations overall were forced labor, and destruction or seizure of food, livestock, or crops. Households with in-migrants were more likely to experience forced labor than households with out-migrants 8.2% vs. 2.2%), though this relationship did not reach statistical significance. Compared to households without migrants, households with out-migrants were less likely to experience destruction of food, livestock or crops (4.2% vs. 8.5%, aPR = 0.49), though the association was not statistically significant. Associations between land confiscation and household in- or out-migration did not reach statistical significance (Table 6).

## Discussion

Migration patterns result from a complex interplay of political, economic, and social factors. There is a need to better understand the determinants and impact of migration, and household surveys represent an important means of gathering this data [28]. This survey presents the first household level data on in- and out-migration in Eastern Myanmar and the associations between migration, health outcomes, and human rights after the political transition. As described in Parmar, et al. 2014 and 2015 [18, 20], a substantial decrease in human rights violations was observed in the region between 2009 and 2013 while health indicators remain poor, including infant and child mortality and access to reproductive health. In this context, several key findings with regards to migration were found.

### Rates of out-migration greatly exceed in-migration

Despite recent political changes, out-migration outstripped in-migration more than 6:1 overall – for international migration this ratio was 29:1. Despite the recent political transition, qualitative increase in political stability in Eastern Myanmar, and the discussion among neighboring governments and United Nations agencies to repatriate immigrants and refugees back to Myanmar, many more people from Eastern Myanmar left the country than returned to their villages in 2012–2013. Internal migrants in Eastern Myanmar who did not cross an international boundary were more likely to move within the same State than to cross State boundaries, for example to work in large cities such as Yangon or Mandalay. This is consistent with findings from a national survey of internal migrants, which found that within-State migration was more common in ethnic border regions than more central locations such as Bago, Mandalay and Yangon, where a greater proportion of migrants had moved across state lines [17]. It is possible that this high level of internal migration has been facilitated by improved internal security, allowing migrants from more impoverished regions to move to urban centers seeking opportunities.

### Migrants driven by employment, education, and family

Opportunities for employment and education appear to be the primary drivers of migration out of the household; reasons involving family or marriage appear to explain most migration into the area. On average, several family members migrated into a household together (mean 2.5) while on average only 1 individual migrated out of a household. The largest proportions of those migrating into the household were children under the age of 15 followed by people over the age of 25 (46.3 and 34.0%, respectively, Table 1). However, among children under 15 and adults over 25, migration out is 3–5 times more common than migration in; and among young adults 15–25 years of age, out-migration away from the rural ethnic villages included in this survey is *10 times* more common than in-migration among women, and nearly *20 times* more common than in-migration among men. As outlined in Table 4, younger and older adults reportedly left these rural communities for reasons related to education and employment; and there is no indication that this substantial efflux of economically productive people from these rural areas was complemented by a reciprocal return among older age groups. Our findings our consistent with well documented process of urbanization in Myanmar [29, 30], and with a relative lack of opportunity in rural areas[31]. The dearth of opportunity in rural ethnic communities may pose challenges for programs that rely on voluntary return of refugees and IDPs to the rural areas they left years-, or decades- in the past [12, 15].

### Migration and health

Reproductive health fared worse in households with in-migration, as women in these households were more likely to be malnourished and had a higher unmet need for contraception. These results are consistent with evidence from Southeast Asia [32–35] but appear to contrast with those of a survey in Myanmar that found a “healthy migrant” effect among migrant women who were more likely than non-migrants to use modern family planning methods and to use antenatal care during pregnancy [36]. Results may have differed due to a lack of overlapping areas, and differential availability of health services in surveyed regions. While women of reproductive age in households with in-migration suffered higher rates of malnutrition, this is not true of children under 5. The reasons for this are unclear, and further study of rates of malnutrition among these populations with reference to migration are needed to determine whether this is a consistent finding.

Out-migration also has effects on those household members left behind. Households with out-migration had a slightly higher number of dependent children, were more likely to be single parent households either with or without children under 5, and the survey respondent was more likely to be at risk for depressive symptoms using the PHQ-2 scale. This aligns with suggestions by some authors that suggest those “left behind” may suffer multiple consequences including higher rates of depression resulting from loss of a primary breadwinner, increased social isolation, or decreased access to health and other services [37, 38]. In contrast, studies by Abas et al. suggest that in some populations, mental health of those who remain improve when a member of the household migrates out, possibly as a result of increased household resources from remittances [39, 40]. Thus, effects of out-migration on those “left behind” is likely multifactorial, resulting from the balance of positive and negative factors at the household level. For example, a higher proportion of out-migrant households had extended family present at the time of the survey, suggesting that broader kinship networks may provide additional support when a family member has left. Supporting this finding, a report highlighted that many grandparents in rural Myanmar are being left to care for one or more children while their parents migrate away to earn money, most often in neighboring Thailand [41].

### Human rights violations and migration

As noted in Table 6, exposure to human rights violations do not appear to be a driver of recent in- or out-migration according to these analyses, and exposure to human rights violations has decreased in this population compared to previous studies [20].

**Table 6:** Migration and Exposure to Human Rights Violations

Household exposures and outcomes	HH with in migrants	HH with out migrants	HH with no migrants	Risk Ratio: IN vs. NON	Risk Ratio: OUT vs. NON
Human Rights Violation	n, % (95% CI)	n, % (95% CI)	n, % (95% CI)	Adjusted RR (95% CI)	Adjusted RR (95% CI)
Forced Labor	46, 8.2% (1.2–15.1%)	128, 2.2% (0.9–3.5%)	1147, 3.7% (1.5–6%)	1.35 (0.66,2.78)	0.61 [0.34,1.08]
Destruction and seizure of food, livestock, or crops	39, 7.5% (0.5–14.4%)	17, 4.3% (1.4–7.2%)	1975, 8.5% (4.2–12.8%)	0.82 [0.35,1.91]	0.49 [0.24,1.02]
Confiscation of land	0, 0.0% (0–0%)	48, 0.5% (0–1.2%)	256, 0.5% (0–1.2%)	0.00 [0.00,0.00]	0.88 [0.38,2.06]
Physical injuries (gunshot, wounds, landmine injuries, beatings, stabbings)	0, 0.0% (0–0%)	8, 0.2% (0–0.6%)	40, 0.2% (0–0.3%)	0.00 [0.00,0.00]	0.62 [0.08,4.90]
Detained or tied up	0, 0.0% (0–0%)	0, 0.0% (0–0%)	10, 0.0% (0–0%)	0.00 [0.00,0.00]	0.00 [0.00,0.00]
Landmine injury in last 15 years	47, 11.4% (3–19.7%)	383, 7.0% (3.3–10.7%)	1071, 5.0% (3.2–6.9%)	1.92 [0.96,3.84]	1.07 [0.76,1.51]
Number of human rights violations experienced by household					
Zero	477, 89.7% (81.6–97.7%)	4603, 92.2% (88.7–95.7%)	21,799, 88.7% (84.4–93%)		
One or more	57, 10.3% (2.3–18.4%)	408, 7.8% (4.3–11.3%)	3062, 11.3% (7–15.6%)	0.83 [0.40,1.74]	0.64 [0.39,1.06]

24 HH with in and out-migration omitted from this analysis

However, human rights violations do continue to shape the landscape of lived experience in Eastern Myanmar in 2012–2013. A history of forced labor in the previous year was reported by more in-migrant (8%) than out-migrant households (2.2%), though this finding did not reach statistical significance. Other than respondent demographics and whether migrants had crossed a state boundary, our survey did not collect more detailed information on several risk factors possibly associated with forced labor in Myanmar such as low income, recent death of a breadwinner, use of a recruiter outside of friends or family (*‘pwe sar’),* or the type or conditions of current employment. The absolute prevalence of forced labor in our study is lower than the very high prevalence of forced labor (26%) documented in 2014–2015 by the International Labour Organization (ILO) in nationally representative sample of 7295 internal migrants. The ILO study documented forced labor among 39.6, 32.3 and 13.1% of migrants to Karen, Karenni and Mon States represented in the present survey in Eastern Myanmar [17]. The ILO study was explicitly designed to document multiple dimensions of forced labor and trafficking, using multiple indicators of involuntariness and coercion ranging from lack of overtime or breaks and withholding identity papers. In contrast, our survey asked a single question about a more narrow definition of forced labor, limited to being “forced to work against [one’s] will by soldiers or authorities,” which may explain the lower prevalence documented in our study.

Although land confiscation was reported by over 300 households in our study area, it does not appear to have been a major driver of out-migration. Households with one or more members who had moved away, and households without migrants, were similarly likely to report a history of land confiscation (0.5%); and among the 48 households with out-migrants that reported a history of land confiscation, only 2 cited land confiscation as the primary reason the household member had left. Land confiscation was more commonly cited by individuals moving into their current residence (4.5% of all in-migrants); and all 37 had moved into a household that had not experienced land confiscation.

### Limitations

It is known that household exposures human rights violations have decreased over the period between 2009 and 2013 in this region. While it is reasonable to assume that this was once a major driver of migration, it is difficult to interpret how the relative changes in security have impacted in- and out-migration at the household level with certainty. Along these lines, it is critical to remember that households that migrated out of Myanmar in their entirety as a result of insecurity or for other reasons are not captured by this study. In addition, the sampling methodology excludes the homeless, or any actively migrating populations along the border regions, which may underestimate the association of migration on studied outcomes [42]. This study did not differentiate between refugees or economic migrants, and did not explicitly capture remittances, which may positively contribute to economic and food security and produce salutary effects on health outcomes among households with out-migrants. Patterns identified herein are specific to the eastern Myanmar region, and would not be expected to apply to areas experiencing active conflict, such as Rakhine, Kachin or Shan states. Finally, all associations are just that—given the methodology used, causality cannot be determined (e.g., out-migration is associated with household respondents screening positive for depressive symptoms, but we cannot say that this relationship is a causal one).

It is important to keep in mind that educational attainment, linguistic, religious and ethnic affiliations were asked of the household respondent only, and not gathered specifically with regards to the migrant. Without directly surveying migrants themselves it is challenging to completely understand the implications of this with regards to bias, however the authors’ collective years of experience support the assertion that households in eastern Myanmar tend to be similar with regards to many of the above stated affiliations. Thus, this represents a source of unmeasured reporting bias that might affect these findings. Additionally, all “reasons for migration” were reported by the respondent, not by the migrant themselves, which may lead to some incorrect attributions. Households with both in- and out-migration were excluded from these analyses (total 24), as the primary goal was to examine trends associated with either in or out migration. These households represented 2% of the total number of households in the sample with any migration, and less than 1% of all households in this sample.

## Conclusion

Despite a decrease in civil conflict and an overall decline in household exposures to human rights violations since the election, migration patterns suggest that conditions in eastern Myanmar continued to be more difficult than neighboring regions. Out-migration was far more common than in-migration. A majority of migration is driven by the pursuit of employment or an education, while a substantial number of individuals continued to move into the study area for marriage and other family-related reasons. Residents left behind appear to experience increased risk of depressive symptoms, and women of reproductive age in households with in-migration were more likely to be malnourished and have an unmet need for contraception.

A recent visit by the authors to the Thai-Myanmar border found that NGOs and ethnic health organizations are struggling to obtain funding needed to provide services to Burmese populations displaced into western Thailand, both in camp and non-camp settings. In this complex political, economic, and social environment, both migration and refugee return must be managed with a careful sensitivity to the remaining relative paucity of education and economic opportunities for citizens of Myanmar, and the impact of migration on health.

Further study is needed with regards to the implications of migration on the health of individual migrants themselves and to understand the experiences of households that have left their homes in their entirety.

## Footnotes

1 For ease of presentation, state/administrative region will be simply referred to has “state” hereafter.
